# Tapping the Role of Microbial Biosurfactants in Pesticide Remediation: An Eco-Friendly Approach for Environmental Sustainability

**DOI:** 10.3389/fmicb.2021.791723

**Published:** 2021-12-23

**Authors:** Aman Raj, Ashwani Kumar, Joanna Felicity Dames

**Affiliations:** ^1^Metagenomics and Secretomics Research Laboratory, Department of Botany, Dr. Harisingh Gour University (Central University), Sagar, India; ^2^Mycorrhizal Research Laboratory, Department of Biochemistry and Microbiology, Rhodes University, Grahamstown, South Africa

**Keywords:** pesticides, bioremediation, biosurfactants, hydrophobic, amphiphilic, metagenomics

## Abstract

Pesticides are used indiscriminately all over the world to protect crops from pests and pathogens. If they are used in excess, they contaminate the soil and water bodies and negatively affect human health and the environment. However, bioremediation is the most viable option to deal with these pollutants, but it has certain limitations. Therefore, harnessing the role of microbial biosurfactants in pesticide remediation is a promising approach. Biosurfactants are the amphiphilic compounds that can help to increase the bioavailability of pesticides, and speeds up the bioremediation process. Biosurfactants lower the surface area and interfacial tension of immiscible fluids and boost the solubility and sorption of hydrophobic pesticide contaminants. They have the property of biodegradability, low toxicity, high selectivity, and broad action spectrum under extreme pH, temperature, and salinity conditions, as well as a low critical micelle concentration (CMC). All these factors can augment the process of pesticide remediation. Application of metagenomic and *in-silico* tools would help by rapidly characterizing pesticide degrading microorganisms at a taxonomic and functional level. A comprehensive review of the literature shows that the role of biosurfactants in the biological remediation of pesticides has received limited attention. Therefore, this article is intended to provide a detailed overview of the role of various biosurfactants in improving pesticide remediation as well as different methods used for the detection of microbial biosurfactants. Additionally, this article covers the role of advanced metagenomics tools in characterizing the biosurfactant producing pesticide degrading microbes from different environments.

## Introduction

Soil pollution and land degradation are global problems originating from anthropological and natural sources (Malla et al., 2018). Urbanization and industrialization are the major sources of anthropological pollution while the use of chemical agents over the year for increasing crop production has led to the spread and accumulation of pollutants in the environment (Prihandiani et al., 2021). The most common contaminants in the soil are heavy metals, polycyclic aromatic hydrocarbons (PAHs), or pesticides. Pesticides are the chemical compounds used to kill unwanted pests such as bugs, flies, rodents, nematodes, fungal pathogens, and unwanted herbs to maintain plant health and increase agricultural production on limited land. Pesticides play an essential part in fulfilling the world food demand, though they are very hazardous, persistent, recalcitrant, and have extended half-life properties (Wattanaphon et al., 2008; Damalas and Eleftherohorinos, 2011; Odukkathil and Vasudevan, 2016; Lamilla et al., 2021). The most common pesticides used in India and the other countries are organophosphates like chlorpyrifos, profenofos, and glyphosate, with a few being organochlorine i.e., mirex, lindane, and chlordane (Lamilla et al., 2021). These organophosphorus and organochlorine pesticides are not target-specific and have high biological stability in the soil and water bodies, polluting the ecosystem and making it pestilent for humans and other organisms such as pollinators, cattle, microbes, and aquatic organisms (Gennari et al., 2009; Sequinatto et al., 2013). Most organophosphate pesticides are classified as class II carcinogens with mutagenic, teratogenic, and carcinogenic effects on humans and other organisms (Bhatt et al., 2021a).

Looking at the grave danger of these toxic pollutants, there is an urgent need to deal with the harmful impacts of these toxic pesticides. Most physical and chemical pesticide removal methods have been in use for a long time. These methods include aeration, oxidation, excavation, incineration, landfilling, and storage, etc., which is labor-intensive, time-consuming, inefficient, and are not considered a sustainable method of remediation because they result in the generation of several secondary pollutants (Yoshikawa et al., 2017; Bhatt et al., 2021a). As a result, the implementation of bioremediation appears as the only answer to the issue mentioned above since it uses the ability of live indigenous microorganisms to clean the polluted site (Singh et al., 2011). Researchers worldwide are now trying to create the most cost-effective and long-term sustainable method for pesticide bioremediation. Microbial biosurfactant-based remediation is a natural, cost-effective, and environmental friendly method of on-site degradation of pesticides and other xenobiotics in which biosurfactants make pesticides bioavailable and microbes use them as a source of carbon, nitrogen, and phosphorous (Abouseoud et al., 2008; Luna et al., 2013; Chaprão et al., 2015; Odukkathil and Vasudevan, 2016; Bhatt et al., 2021b; Lamilla et al., 2021).

**FIGURE 1:**
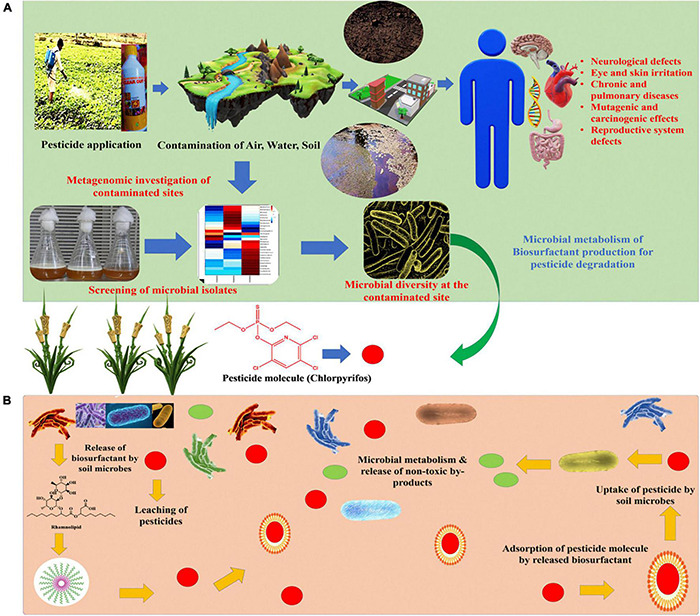
**(A)** Shows the pesticide application in the field and its fate in the environment leading to contamination of air, water, and soil along with screening and isolation of microbes residing at the contaminated site for biosurfactant production **(B)** presented the mechanism of biosurfactant mediated pesticides degradation.

Biosurfactants are secondary metabolites produced by microorganisms used in many commercial applications due to their low toxicity, substantial biodegradability, and environmentally benign nature (Thavasi et al., 2011; Chaprão et al., 2015; Javee et al., 2020). Biosurfactants consist of both hydrophilic and hydrophobic regions that are formed from amino acids, as well as saturated and unsaturated fatty acids, respectively (Sharma et al., 2015; Bhatt et al., 2021b), which enables a reduction in surface tension and reaching out between two solvate molecules, thus accelerates the solvation of hydrophobic molecules in aqueous media for emulsion formation (Banat, 1995; Lamilla et al., 2021). It has been observed that hydrocarbon-contaminated areas are the best places to isolate biosurfactant-producing microorganisms to improve pesticide remediation (Singh et al., 2016; Tan and Li, 2018; Bhatt et al., 2021a; Lamilla et al., 2021). Figure 1 depicted the entry of pesticides into the food chain and the fate of pesticides and presented the mechanism of biosurfactant mediated pesticides degradation. Several published reports are available wherein biosurfactant producing potential of microbes have been utilized for bioremediation of pesticides.

Although most of the studies on bioremediation are done using single microbes (particularly culturable ones). Several reports suggest the microbial consortia enhances the remediation process compared to a single isolate (Pacwa-Płociniczak et al., 2011; Rasheed et al., 2020; Madamwar et al., 2021). While in several cases, the role of unculturable microbes is often ignored, and the underlying mechanism of microbes-mediated pesticides degradation is still unexplored (Singh and Gupta, 2018; Singh et al., 2019). The use of metagenomics to understand the underlying mechanisms of biodegradation in *in-situ* and to forecast degradation potential has yet to be studied. So, there is a lack of information about microbes’ functional genes and genetic potential and their products like biosurfactants involved in degradation (Datta et al., 2020; Femina Carolin et al., 2020). Against this background, this article aims to give a comprehensive overview of the function of microbial biosurfactants in the remediation of pesticides. We also briefly addressed how improved metagenomics techniques might assist clean-up by providing access to uncultivable microbial species.

## Emerging Pesticide Pollution: A Global Concern

The tremendous demand to produce food at reasonable prices has forced farmers/growers to use chemical fertilizers and pesticides (Sarath Chandran et al., 2019). Organochlorine pesticides such as DDT (dichlorodiphenyltrichloroethane) and Gammaxene were used extensively during the second world war, but they proved to be an ecological disaster for the world and was banned by the United States in 1972 due to their toxic effects on the peripheral nervous system and its non-biodegradable nature. Several developed and developing nations banned the use of most organochlorine pesticides, including DDT (Mansouri et al., 2017; Peng et al., 2020). This led to increased demand for organophosphate pesticides such as malathion, parathion, monocrotophos, etc., due to their wide action spectrum and moderate toxicity (Narenderan et al., 2020; Parks et al., 2021). However, feeding the exponentially growing population on declining land area forced farmers to use these pesticides more than the recommended dose i.e., 1 U.S liquid pints per acre of land or 0.473 liters per acre of land area (Tchounwou et al., 2015; As per U.S. Environmental Protection Agency | US EPA, 2021). Insecticides, herbicides, rodenticides, and fungicides are among the most regularly used pesticides (Malla et al., 2018) and were used excessively during the green revolution to increase productivity and reduce crop loss (Sarath Chandran et al., 2019). Pesticide use is rising and negatively influencing the environment, particularly the soil quality and health (Rawat et al., 2020), as only 1% of sprayed pesticides kill target species; the rest pollute the ecosystem by interacting with soil and generating more complex metabolites. For example, chlorpyrifos produces 3,5,6-trichloro-2-pyridinon (TCP), an antimicrobial metabolite that kills beneficial soil microorganisms and due to its eco-toxicity, the United nation banned this pesticide in the year 2020. However, chlorpyrifos is extensively used in developing countries like India, Bangladesh, and Pakistan (John and Shaike, 2015; Shabbir et al., 2018; Kalyani et al., 2021). Pesticides strongly adsorb the soil’s organic matter, which restricts its desorption. Most of the pesticides are non-polar compounds having hydrophobic properties and are insoluble in water (Bhatt et al., 2021b; Kalyani et al., 2021). Pesticides are highly recalcitrant on exposure to humans; they lead to several disorders related to the central nervous system as most of these chemicals inhibit acetylcholinesterase receptor activity, causing nerve damage. Apart from it, inhalation of pesticides leads to several respiratory disorders and these chemicals also have mutagenic and carcinogenic potential causing disorders related to fertility, excretory system, skin, and eye defects (Figure 1; Foong et al., 2020; Giri et al., 2020). According to reports on poisoning and the impact of synthetic chemicals on human health, numerous cases of intoxication of farmers, rural workers, and their families have occurred during pesticide applications. Unintentional poisonings kill an estimated 3,55,000 people annually and are related to excessive exposure and improper use of hazardous substances (Nayak et al., 2020).

### Pesticide Defilement Status: Indian Context

**FIGURE 2:**
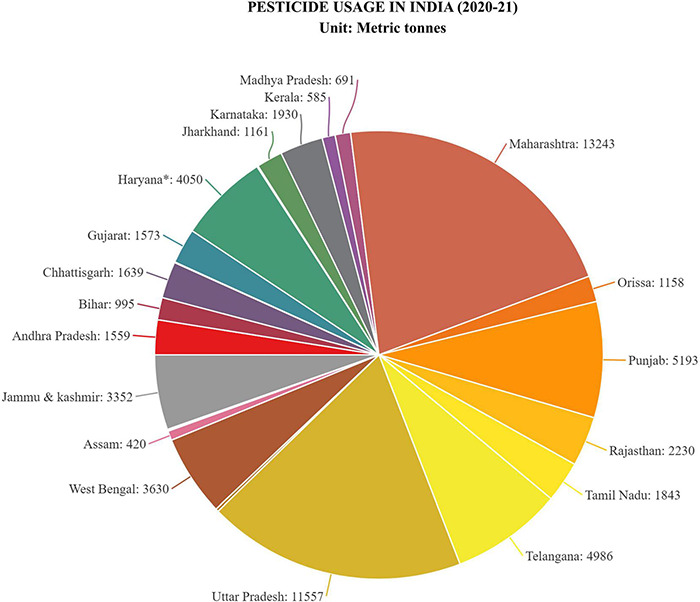
Pesticide use in India (2020–21), [Unit: Metric tons (M.T)]. Pie-chart representing the average usage of pesticide by different Indian states in the year 2020–21. Highest usage among the states for which the pie-chart is made has been noted for Maharashtra with average usage of 13,243 M.T. while lowest for Andaman and Nicobar island with 1 M.T. Data were taken from statistical database of government of India, directorate of plant protection, quarantine and storage (*Statistical Database | Directorate of Plant Protection, Quarantine and Storage |* GOI, 2021).

Agriculture and allied sectors provide a living for most of India’s population (57%) (Hobbs et al., 2009). India stands second in pesticide consumption (0.29 kg/ha) among all Asian continents (Sharma et al., 2020; FAOSTAT, 2021). Crop production in India fell short of the country’s demand in the post-independent period (Sebby, 2010). The implementation of the green revolution revolutionized India’s conventional comestible farming into capital intensive, modernized, surplus-producing agriculture, resulting in a 10-fold increase in overall food grains production between 1960 and 2000 (Davies, 2003; Kannuri and Jadhav, 2018). High yielding varieties (HYVs) were deployed as a part of the green revolution and these HYVs relied on enormous amounts of nitrogenous fertilizers to provide the desired crop outputs to feed the ever-expanding India’s population (Kannuri and Jadhav, 2018; Sharma et al., 2019; Mishra et al., 2020). Narrow heritable traits of high yielding varieties of rice and wheat, as well as monocropping and tropical Indian climate, resulted in significant susceptibility to pests and diseases, but persistent pesticide application resulted in pest resistance. This lead to an over-reliance on pesticides to reduce crop loss, leading to a dramatic increase in pesticide use in India i.e., from 154 metric tons in 1954 to 88,000 metric tons in 2000, a 570 per cent higher in less than a half-century (Kumar et al., 2016; Bonvoisin et al., 2020). However, strict action was taken by the Indian government lead to a decline in pesticide consumption by the year 2015–16 to about 58,634 metric tons from 88,000 metric tons in 2000, but this figure is steadily increasing and has reached about 62,193 metric tons in the year 2020–21 (Figure 2) which is a real cause of concern (Gunnell and Eddleston, 2003; *Statistical Database | Directorate of Plant Protection, Quarantine, and Storage |* GOI, 2021).

An investigation carried out by a group of researchers on the Thamirabarani river system of southern India reported the bioaccumulation of organochlorine pesticides such as aldrin, dieldrin, endosulfan, endrin, and heptachlor in surface water, sediments and fishes and other aquatic flora and fauna. Organochlorines were detected with the help of GC-MS following QuEChERS protocol extraction and were in a concentration ranging from 0.001 to 34.44 μg/l^–1^ in surface waters to as high as 40.46–65.14 μg kg^–1^ in different organs of fishes (Arisekar et al., 2018). Not only vegetables or crop plants are detected with pesticide residues. Even milk samples have been detected with traces of pesticides such as DDT, HCH, endosulfan, and pyrethroids in the Kolkata and Nadia region of West Bengal, India (Kumar et al., 2016; Anand et al., 2021; Ravula and Yenugu, 2021).

The Punjab region of India is highly affected by pesticide poisoning (Figure 2). In a study, out of 111 samples of human blood, 35% of the samples were detected with traces of pesticides like DDT, HCH, profenofos, monocrotophos etc. some samples were detected with a high level of 34.90 ng ml^–1^ (Sharma et al., 2020). Looking at the pesticide contamination status in India, there is an urgent need to develop techniques to deal with these toxic pollutants. Microbial biosurfactant based bioremediation seems to be the most sustainable way to eliminate these toxic compounds from soil and water bodies and to regain soil health.

## Biosurfactants: The Next Age Compounds in Pesticide Remediation and Their Types

Biosurfactants which are surface-active molecules produced naturally by microbial metabolism have gained popularity in recent times. In recent years, there has been a steady increase in the number of research papers focused on the isolation, characterization, and optimization of biosurfactants producing microbes (Das and Kumar, 2018; Olasanmi and Thring, 2018; Khanna and Pattnaik, 2019; Rawat et al., 2020). Biosurfactants are the type of “glycoconjugates (combination of glycoprotein and glycolipids),” and the study of its structure, function, and interaction with the living system is called “glycobiotechnology” (Messner et al., 2013; Enaime et al., 2019). Microbes produce some extracellular biosurfactants such as rhamnolipids, sophorolipids and exopeptidases, and glycol-lipopeptides. A wide variety of anionic and non-ionic synthetic surfactants (Triton X-100, tween-80, tergitol NP10, brij35, sodium dodecyl sulfate etc.) are in use for a long time to accelerate microbial activity whether in the area of xenobiotic remediation or biofuel production (da Rocha et al., 2010).

Furthermore, they are extensively used by pesticide industries as an emulsifiable concentrate in the pesticide formulation. However, synthetic surfactants are highly toxic, non-biodegradable, have low selectivity, high CMC value, and show antimicrobial activity (Bustamante et al., 2012; Yañez-Ocampo et al., 2017). Therefore, another reason for the popularity of biosurfactants is their advantages over their chemically manufactured counterparts, such as having a simpler structure than the synthetic equivalents, being environmentally friendly, and lesser toxicity (Rawat et al., 2020). On the other hand, biosurfactants can survive up to 10% salinity, but synthetic surfactants cannot. Other than their essential role in pesticide remediation (Tan and Li, 2018), they are also used in various commercial products, including medications, cosmetics, cleaning agents, and the food sector (Santos et al., 2016).

**FIGURE 3:**
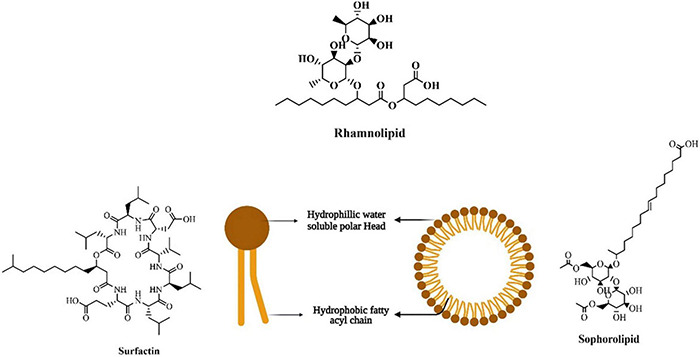
Structure of biosurfactant.

**FIGURE 4:**
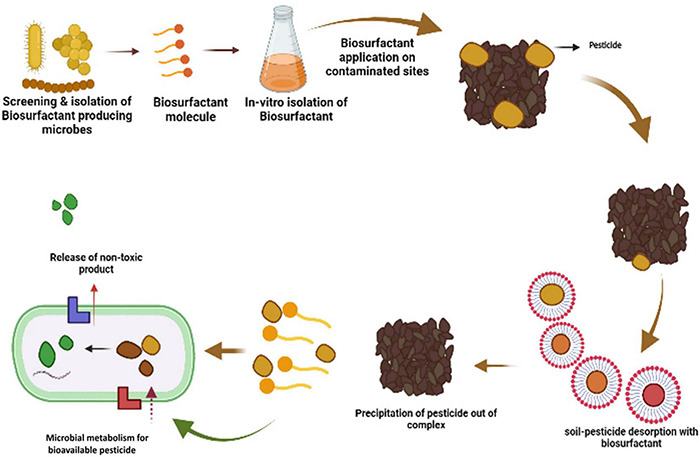
*In-vitro* isolation of biosurfactant and its application at pesticide-contaminated sites. Further steps indicate the adsorption of biosurfactant with the soil-pesticide complex leading to desorption of pesticides from the soil particles. Microbial surfactants precipitate from the pesticide-biosurfactant complex, making pesticides bioavailable for the microbes for their further degradation.

Most microbes produced biosurfactants on their cell surface (amphiphilic molecules) as secondary metabolites at their stationary phase of growth (Soberón-Chávez et al., 2005; Moya Ramírez et al., 2015). These biosurfactants include glycoproteins, glycolipids, glycopeptides, glycosides, peptidoglycan, and lipopolysaccharides, which are the diverse forms of glycoconjugate-based biosurfactants (Varjani and Upasani, 2016; Bhatt et al., 2021b). One of the distinguishing features of biosurfactants is the hydrophilic-lipophilic balance (HLB), which determines the proportion of hydrophilic and hydrophobic elements in surface-active substances (Pacwa-Płociniczak et al., 2011). Biosurfactant activities are dependent on the concentration of surface-active molecules till the critical micelle concentration (CMC) is attained. Biosurfactant compounds form micelles, bilayers and vesicles at a concentration above CMC (Rasheed et al., 2020) and these micelles can reduce surface and interfacial tension and increase the solubility and bioavailability of hydrophobic pesticide molecules (Bhatt et al., 2021b). Surfactant efficiency is frequently measured using the CMC, as efficient biosurfactants have a low CMC, and require less biosurfactant to reduce surface tension (Pacwa-Płociniczak et al., 2011; Bhatt et al., 2021a,b). Micro-organisms may synthesize biosurfactants from various carbon sources, but *Glycine max*, *Zea mays, Brassica napus*, and *Olea europaea* can be employed to increase output (Bhatt et al., 2021b). Many researchers have tried producing biosurfactants from unconventional and agricultural-based raw materials; however, this approach has not yet been commercialized. These lipopeptides may be made from low-cost raw materials such as *Saccharum officinarum*, *Zea mays*, molasses, agricultural wastes, and others that are easily accessible in large numbers to be cost-effective (Hippolyte et al., 2018; Domínguez Rivera et al., 2019; Rawat et al., 2020). The structure and composition of the biosurfactant molecule and the role of biosurfactants in pesticide remediation is shown in Figures 3, 4.

### Types of Biosurfactants

The majority of biosurfactant is either neutral or anionic while cationic biosurfactants are possessing amine groups. Long-chain fatty acids make up the hydrophilic moiety, which can be any amino acid, glycogen, cyclic peptide, alcohol, or phosphate carboxyl acid, whereas monosaccharides, proteins, polysaccharides, or peptides make up a hydrophobic portion of the biosurfactant (Saharan et al., 2011; Bhati et al., 2019). Biosurfactants typically have a molar mass of 500–1,500 Dalton (Akbari et al., 2018). These are generally classified as per their chemical structure and microbiological derivation and are as follows:

The bulk of biosurfactants are glycolipids, carbohydrates with an ester group that connects them to long-chained aliphatic acids or hydroxyl aliphatic acids. Rhamnolipids, trehalolipids, and sophorolipids are well-known glycolipids (Rawat et al., 2020). Apart from glycolipids, lipopeptides, phospholipids, fatty acids, polymeric, and particulate biosurfactants are the other common types (Mnif and Ghribi, 2016; Kapoor et al., 2019). Figure 3 shows the structure and composition of the biosurfactant molecule.

**Rhamnolipid** consists of one or two rhamnose molecules linked to one or two hydroxyl decanoic acid molecules (de Oliveira Schmidt et al., 2021; Figure 3). *Pseudomonas aeruginosa* is said to produce the most rhamnolipids of any known microbial species, followed by *Burkholderia* species (Chong and Li, 2017; Ramirez et al., 2020). Recently, *Marinobacter* species and *Pseudomonas mendocina* have been isolated from the marine environment and are an excellent producer of rhamnolipid (Tripathi et al., 2019; Twigg et al., 2019). **Trehalolipids** are extensively produced by *Rhodococcus, Mycobacterium, Nocardia*, and *Corynebacterium* species (Williams and Trindade, 2017; Rawat et al., 2020). *Arthrobacter* species and *Rhodococcus erythropolis* are reported to produce trehalolipids that are non-toxic, versatile, and can reduce surface and interfacial tension in the culture broth. Trehalose lipids have been found to have enhanced surfactant activity in various situations and have been studied extensively (Williams and Trindade, 2017; Rawat et al., 2020). **Sophorolipids** are produced by non-pathogenic yeast species and are produced in large quantities (400 g L^–1^). These are made up of the glycosidic connection between sophorose, a dimer form of glycogen joined by β-1,2 linkage and a long-chain hydroxy fatty acid (Figure 3). Many applications prefer the lactone form of sophorolipids, which comprises at least 6–9 different hydrophobic sophorolipids. *Candida bombicola, Pseudomonas aeruginosa* M408, and *Starmerella bombicola* are well-known sophorolipid producers (Rawat et al., 2020). **Lipopeptides** are polypeptide chains with varying lengths of β-hydroxy fatty acid and non-polar tails connected to them. *Bacillus* and *Pseudomonas* species are the most investigated lipopeptide producers, while *Bacillus subtilis* produces surfactin (Janek et al., 2021), the most potent lipopeptide known (Kumar et al., 2021a). Lipopeptides are the type of natural product produced by non-ribosomal peptide synthases (NRPS) (De Giani et al., 2021), which are huge multifunctional enzyme clusters (Williams and Trindade, 2017; Lamilla et al., 2021). **Surfactin** is considered one of the most potent biosurfactants (Meena et al., 2021) composed of cyclic lipopeptides with seven amino acid ringed structures linked to a fatty acid chain through lactone linkage (Figure 3; Onaizi, 2018; Sarwar et al., 2018). Table 1 summarizes the type of biosurfactant as well as the microbes that produce them and their roles in pesticide remediation. Fatty acids, Phospholipids, and Neutral lipids may be formed by a diverse group of microbial species that grow on various substrates, including n-alkanes (Varjani et al., 2021). *Thiobacillus thioxidans* is a well-known producer of phospholipids and has been reported to reduce sulfur elements from the soil. *Corynebacterium lepus* produces corynomycolic acid, which helps lower surface and interfacial tension at varied pH (Kosaric et al., 2018). Polymeric biosurfactants such as liposan, emulsan, alasan, lipomanan are the most investigated polymeric biosurfactants. *Acinetobacter calcoaceticus* RAG-1 produces an emulsan type of polymeric surfactant, which helps in emulsifying hydrocarbons in water (Uzoigwe et al., 2015). Liposan is synthesized by *Candida lipolytica* (Panjiar et al., 2017).

**TABLE 1:** Microbial biosurfactants and their role in pesticides degradation.

Microorganism	Biosurfactant Produced	Substrate for Production	Pesticide Degraded	Concentration of Pesticide	Degradation (%)	Identification technique	References
*Pseudomonas, Rhodococcus*	Rhamnolipid	Vegetable oil waste, *Zea mays* waste	Cypermethrin, Chlorpyrifos	2%w/v	8–63%, 39–56%	Emulsification, FTIR, TLC, MALDI-TOF	Nitschke and Pastore, 2006; Aguila-Torres et al., 2020; Lamilla et al., 2021
*Pseudomonas* sp.	Rhamnolipid	Animal waste	Chlorpyrifos	0.01 g l^–1^	98%	Gas chromatography-mass spectrometry (GC-MS/HPLC)	Singh et al., 2009; Lima et al., 2011
*Pseudomonas aeruginosa* CH7	Rhamnolipid	Cassava flour wheat	β- cypermethrin	25–900 μg L^–1^	90%	Mass spectrometry	Zhang et al., 2011; de Andrade et al., 2016
*Arthrobacter globiformis*	Rhamnolipid	Agro-industrial waste	DDT	0.04 mg/L	65%	**−**	Bai et al., 2017; García-Reyes et al., 2018
*Pseudomonas aeruginosa*	Rhamnolipid	Canola oil, Agro-industrial waste	Endosulfan, Quinalphos	320 mg/L, 10,000 mg/L	90%, 94%	FTIR/TLC Spectrophotometer	Abbasi et al., 2012; Nair et al., 2015; Pérez-Armendáriz et al., 2019; Briceño et al., 2020; Bhatt et al., 2021b
*Pseudomonas* sp. chlD	Rhamnolipid	Sunflower oil waste	Chlorpyrifos	10 mg/L	99%	FTIR spectra analysis	Kaskatepe and Yildiz, 2016; Singh et al., 2016; Shabbir et al., 2018
*Lysinibacillus sphaericus* IITR51	Rhamnolipid	Soybean waste oil	Endosulfan and HCH	50 and 100 mg/L	>solubility	**−**	Bhatt et al., 2019; Gaur et al., 2019; Lamilla et al., 2021
*Pseudomonas aeruginosa and Sphingomonas* sp.	Rhamnolipid	Agro waste	Hexachlorocyclohexane (HCH)	40 mg/L	95%	FTIR, Emulsification, GC-MS	Manickam et al., 2012; Das and Kumar, 2018; Niu et al., 2019
*Rhodococcus* sp. IITR03	Trehalolipid	Soybean oil waste	Dichlorodiphenyltri chloroethane (DDT)	282 μM	60%	LC-MS (Liquid chromatography-MS) and chemical analysis	Bages-Estopa et al., 2018; Soares da Silva et al., 2019; Bhatt et al., 2021a
*Burkholderia cenocepacia* BSP3	Glycolipid	Frying oil waste	Parathion	500 mg/L	Enhanced solubility	FTIR/chemical analysis	Wattanaphon et al., 2008; Schultz and Rosado, 2020
*Pseudomonas* sp. B0406	Glycolipid	Soybean waste oil	Methyl parathion	**−**	>solubility	LC-MS, FTIR	Cortés-Camargo et al., 2016; Patowary et al., 2017; García-Reyes et al., 2018
*Serratia marcescens* UCP-1549	Lipoprotein	Cassava wastewater	Organic pollutants	**−**	**−**	Mass spectrometry	Casullo De Araújo et al., 2010; de Andrade et al., 2016
*Paenibacillus* sp. D9	Lipopeptide	Soybean oil waste	HCH	**−**	49–65%	TLC/FTIR Thin-layer chromatography/Fourier transform infrared spectroscopy, Affinity chromatography	Li et al., 2016; Jimoh and Lin, 2019b; Marcelino et al., 2019
*Bacillus subtilis* MTCC 1427	Lipopeptide	Soybean oil waste	Endosulfan	400 μg/ml	100%	TLC/IR	Bhatt et al., 2021b
Consortia of *Bordetella petrii* IGV 34 and *Bordetella petrii* II GV 36	Unidentified biosurfactant	**−**	Endosulfan	3,400 mg/L	100%	**−**	Odukkathil and Vasudevan, 2016
*Pseudomonas aeruginosa* B1, *P. fluorescens* B5, *P. stutzeri* B11 and *P. putida* B15	Exopolysaccharides	Saw dust	2,4-D	0.2% v/v	70%	HPLC	Onbasli and Aslim, 2009
*Bacillus algicola, Rhodococcus soli, Isoptericola chiayiensis*	Rhamnolipids	Potato process effluent, corn steep liquor	Crude oil	**−**	65%	FTIR, LC-MS, GC-MS	Sachdev and Cameotra, 2013; Lee et al., 2018
*Actinomycetes, Bacillus, Pseudomonas, Rhodococcus*	Lipopeptide, sophorolipid, glycolipid	Date molasses	Organic pollutants	**−**	63–84.6%	Lyophilization, Pedant drop method	Al-Bahry et al., 2013; Jimoh and Lin, 2019a
							

The biosurfactants listed above are representative of the main types of biosurfactants. Aside from that, many other kinds of biosurfactants and their uses will be described in detail in the next section concerning their application in pesticide remediation.

### Methods for Detection of Microbial Biosurfactants

The discovery of new surfactant-producing microbial strains necessitates advanced microbial screening methods that should be both fast and reliable. In practice, employing a single screening approach for choosing biosurfactant-generating microorganisms has proved to be challenging to get reliable and consistent findings since biomolecules have a wide range of structural and functional characteristics (Adetunji and Olaniran, 2021). Therefore, several screening methods must be employed in parallel to pick a large number of biosurfactant synthesizers from a population of isolated bacteria to get the best results. These techniques are based on the surface tension or emulsification activity of the surfactant, and some of these methods are described in detail in the following section.

#### Measurement of Surface Tension/Interfacial Measurement

This is the most efficient and reliable method for screening microorganisms for biosurfactant production (Adetunji and Olaniran, 2021). Surface tension measures free energy per unit area at an interface or surface (Walter et al., 2010). The stalagometric method, Wilhelmy plate method, du-Nuong-ring method, pendant drop shape method, and axisymmetric drop shape analysis is used to measure the surface tension of culture supernatants directly using a tensiometer (Dusane et al., 2010; Satpute et al., 2010). Distilled water (DW) has a surface tension of 72 mN/m. When biosurfactants are added to the DW, its surface tension is reduced. The ability of biosurfactants to reduce the surface tension of DW to less than 40 mN/m determines its effectiveness. The surface tension of water was reduced to around 30 mN/m by adding a rhamnolipid biosurfactant released by *Pseudomonas aeruginosa* (Dusane et al., 2010; Geetha et al., 2018; Adetunji and Olaniran, 2021).

#### Drop Collapse Method

It is one of the fastest and most straightforward techniques to conduct since it does not need specialized equipment and can be completed with a small sample (Jain et al., 1991). In this technique, surfactants are used to destabilize liquid droplets. On an oil-coated solid surface, drops of culture supernatant or cell suspension are dropped onto the surface. As long as the liquid does not include any surfactants, the polar water molecules are repelled from the hydrophobic surface, and the droplets do not become unstable (Walter et al., 2010). The spread or even collapse of the liquid drop occurs due to the reduction in force or interfacial tension between the liquid drop and the hydrophobic surface when the liquid includes surfactants. The surfactant concentration affects the stability of drops, which is linked to the surface and interfacial tension (Adetunji and Olaniran, 2021). However, despite its speed and simple procedure, this method has low sensitivity because a substantial concentration of surface-active chemicals is required to cause the aqueous drops to collapse on the oil or glass surface (Youssef et al., 2004; Batista et al., 2006; Yu and Huang, 2011).

#### CTAB Agar Plate Method

Extracellular glycolipids or other anionic surfactants can be detected using a CTAB (cetyltrimethylammonium bromide) agar plate method, a semi-quantitative screening method (Hazra et al., 2011). Siegmund and Wagner were the ones who developed this CTAB agar method for the detection of biosurfactant synthesizing microbes (Siegmund and Wagner, 1991). The microorganisms of interest are grown on light blue mineral salt, agar plate containing the cationic surfactant CTAB and the basic dye methylene blue. When the microbes release anionic surfactants on the plate, they combine with CTAB and methylene blue to generate a dark blue, insoluble ion pair (Rajesh et al., 2017). As a result, dark blue halos surround surfactant-producing microbes (Adetunji and Olaniran, 2021). This method is simple, selective for anionic surfactants, and can be performed on agar plates or liquid broth with various substrates and temperatures. Still and all, CTAB is toxic and prevents the growth of several bacterial colonies (Walter et al., 2010).

#### Oil Spreading Assay

In the oil spreading method, crude oil (10 ml) is added to distilled water (40 ml) in a Petri plate, resulting in a thin layer of oil (Alyousif et al., 2021). Following that, culture supernatants (10 ml) are introduced to the oil-water interface (Soltanighias et al., 2019; Dayamrita et al., 2020). The presence of surfactant in the culture supernatant is demonstrated by the displacement of oil and the emergence of a clear zone. Surfactant activity is proportional to the diameter of the clear zone on the oil surface (Sarwar et al., 2018; Adetunji and Olaniran, 2021). The oil spreading method is a quick, accurate and dependable way to identify the synthesis of biosurfactants by a variety of microbes (Walter et al., 2010; Hazra et al., 2011; Hasanizadeh et al., 2017).

#### Penetration Assay

This assay is based on the color shift that occurs when two insoluble phases come into contact. For this experiment, the wells of 96 well micro-plate are filled with 150 μl of a hydrophobic paste made up of oil and silica gel. 10 μl of oil is poured over the paste. The culture’s supernatant is then dyed by adding 10 μl of a red staining solution to 90 μl of supernatant. The colored supernatant is applied to the paste’s surface (Maczek et al., 2007). The hydrophilic liquid will break through the oil film barrier into the paste if a biosurfactant is present. Within 15 min, the silica will shift from more apparent red to cloudy white as it enters the hydrophilic phase. The described effect is based on the fact that when biosurfactants are present, silica gel transitions from hydrophobic to the hydrophilic phase faster. The supernatant without biosurfactants will become hazy but remains red to crimson red (Walter et al., 2010; Singh and Sedhuraman, 2015; Touseef and Ahmad, 2018).

Apart from it, there exist several other methods for detection of biosurfactants producing microbes such as microplate assay (Walter et al., 2010), emulsification capacity assay (Cooper and Goldenberg, 1987), BATH (bacterial adhesion to hydrocarbon) assay (Rosenberg et al., 1980; Nayarisseri et al., 2019; Dayamrita et al., 2020), salt aggregation assay (Ismail et al., 2018), and blood hemolysis assay (Adetunji and Olaniran, 2021).

## Application of Biosurfactants and Their Mechanism of Action in Pesticide Remediation

**FIGURE 5:**
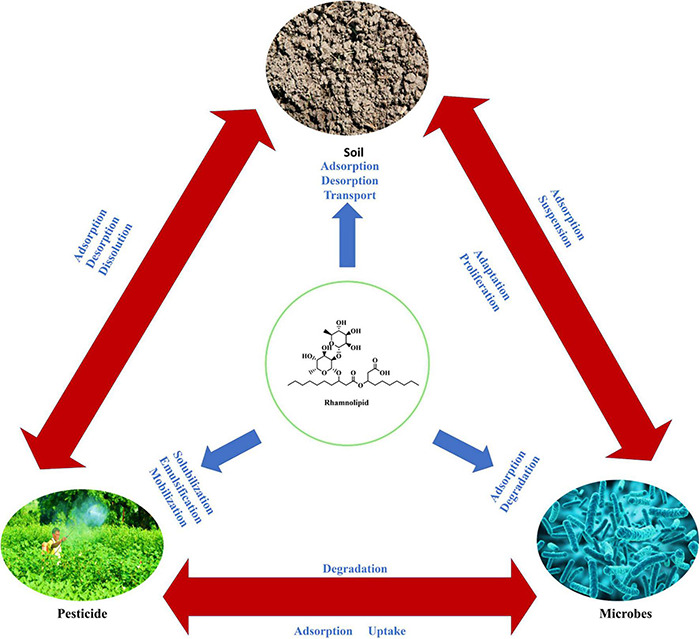
Interaction of biosurfactant with pesticides and the microbes.

Pesticide contamination is a significant problem. The use of biosurfactants for pesticide biodegradation has recently gained popularity. According to ZION market research and global market insights, the worldwide biosurfactant industry is projected to reach $2.4 billion by 2025 (Lai et al., 2009; Sun et al., 2015). The biosurfactant market is anticipated to grow as the pesticide business grows and consumers become more health-conscious (Rawat et al., 2020). The most crucial role that biosurfactants play is the dissociation of toxic pesticide molecules from the soil or water molecules, thus making it bioavailable for the microbes to speed up the remediation process (Figure 5; Inakollu et al., 2004; Whang et al., 2009; Rasheed et al., 2020). Desorption from soil particles leads to a reduction in surface tension, thus enhancing the mechanism of degradation (Singh et al., 2007; Twigg et al., 2019). The probable interaction used for pesticides bioremediation by biosurfactants includes electrostatic interactions, counter-ion binding, ion exchange, and precipitation-dissolution (Banat et al., 2010; Patowary et al., 2017; Xu et al., 2018).

Biosurfactants enhance the surface area of hydrophobic pesticides, increasing their solubility in soil and water by inducing emulsification of pesticide molecules (Bhatt et al., 2021b). The thumb rule of bioremediation is that the more the amount of pesticide that is water-soluble, the greater the amount of pesticide bioavailable to microorganisms. Surface-active apolar flocculating molecules such as biosurfactants, which produce emulsions at and above their critical micellar concentration, may enhance the separation of hydrophobic pesticides from the aqueous phase by creating emulsions at and above their critical micellar concentration (CMC). When pesticides are released into the environment, they become more bioavailable to possible degraders, which may help alleviate the worry about pesticide contamination of soil and water bodies (Zhou et al., 2011; Moya Ramírez et al., 2015). As a result, the soil becomes free of pollutants, productive, and suitable for crop cultivation (Fenibo et al., 2019; Jimoh and Lin, 2019a). The overall mechanism of soil, microbes and pesticide interaction is shown in Figure 5.

Rhamnolipids are the most widely used biosurfactants in industrial and environmental clean-up applications. The potential of rhamnolipid in bioremediation has been extensively studied in *Pseudomonas* and *Burkholderia* species (Varjani and Upasani, 2016). Rhamnolipids obtained from *Pseudomonas aeruginosa* enhance biodegradation of herbicide trifluralin and insecticide chlorpyrifos in the contaminated soil-water bodies (Singh et al., 2016; Tan and Li, 2018). It has been reported that the presence of glycolipid type of biosurfactant obtained from *Pseudomonas* species enhances solubilization of methyl parathion and endosulfan (García-Reyes et al., 2018). With around 100 gL^–1^, *Pseudomonas aeruginosa* is regarded as the top rhamnolipid producer and it produces two forms of rhamnolipids in liquid suspension i.e., mono and di-rhamnolipid (Varjani and Upasani, 2016) by rhamnosyl transfer enzymatic reaction with the help of rhamnosyltransferase enzyme (Soberón-Chávez et al., 2005; Varjani and Upasani, 2017). The hydrophobic and hydrophilic components of the rhamnolipid are formed due to a series of enzymatic processes that take place in microbes. After synthesis, the two halves of the lipid are linked together to form mono- and di-rhamnolipids, respectively (Bhatt et al., 2019). A rhamnolipid was formed from an axenic culture of *Pseudomonas putida* strain DOT-T1E, which aided in the bioremediation of chlorinated phenols (Maia et al., 2019). The trapping of the chlorophenol in the biosurfactant micelles, as well as the hydrophobic connection between these two types of molecules, are at the heart of this action. Likewise, actinobacteria-formed biosurfactant accelerates the bioremediation of xenobiotics (Sponza and Gok, 2011). In the bioremediation of carbendazim with *Rhodococcus* species D-1, rhamnolipids were found efficient. With the highest bioremediation efficiency, the rhamnolipid altered carbendazim degradation in a concentration-dependent manner. It aided carbendazim transesterification and favorable cell surface modification, allowing it to enter *Rhodococcus* species D-1 cells, which were degraded (Bai et al., 2017). Glucolipid type of biosurfactant produced by *Burkholderia cenocepacia* BSP3 complements the solubilization of pesticide (Bustamante et al., 2012). Biosurfactants that spontaneously break down the pesticides are good for the environment and are considered environmentally benign (Jezierska et al., 2019).

Rhizospheric bacteria’s have been reported to play a key role in the degradation of pesticides, accelerating the breakdown as seen during biosurfactant biosynthesis (Bordoloi and Konwar, 2009; Singh, 2015; Dos Santos and Maranho, 2018). The amount of biosurfactant is also vital for microbial development. High quantities of these biosurfactants inhibit microbial growth and breakdown. These findings may not apply to all microbial strains. A study shows that biosurfactant addition increased 30% endosulfan degradation with the help of *Bacillus subtilis* MTCC 1427 in the soil and aqueous solution (Zhou et al., 2011). Endosulfan isomers were found to have more significant mobilization and accessibility in the presence of biosurfactant, which could be due to pesticide solubilization or improved attraction for micro-organism cells. Due to the formation of rhamnolipids by *P. aeruginosa*, the soil adulterated with endosulfan showed accelerated degradation after 7 days of the experiment (Madamwar et al., 2021). The published article indicated that the *Pseudomonas* strain BO406 produced a raw extract of a biosurfactant (glycolipid) that aided in the solubilization of endosulfan (García-Reyes et al., 2018). The strain of *Lysinibacillus sphaericus* IITR51 was used by researchers to develop a thermostable rhamnolipid biosurfactant capable of increasing the solubility of the highly hydrophobic pesticides such as endosulfan and HCH (hexachlorocyclohexane) (Manickam et al., 2012; Gaur et al., 2019). A strain of *Pseudomonas* SB can produce a biosurfactant that enhances DDT breakdown. Rhamnolipid has been reported to increase DDT degradation by 64% from 52% without rhamnolipid (Bhatt et al., 2021b). Studies on mixed consortia of *Pleurotus ostreatus* (white-rot fungus), *Bacillus subtilis*, and *P. aeruginosa* have produced biosurfactants that improve DDT biodegradation (Purnomo et al., 2017; Bhatt et al., 2021b). The hydrophobic herbicide 2,4,5-trichlorophenoxy acetic acid was degraded with the help of a biosurfactant produced from *Pseudomonas cepacian* (Abdul Salam and Das, 2013; Pang et al., 2020; Rawat et al., 2020). Similarly, introducing rhamnolipid to *Rhodococcus* species-D1 resulted in increased carbendazim biodegradation. The addition of rhamnolipid to the soil resulted in around 24–35% biodegradation of trifluralin (Bai et al., 2017). Under atrazine biodegradation, a marine strain of *Bacillus velezensis* MHNK1 produced surfactin lipopeptide. The atrazine was degraded entirely after using a combination of *B. velezensis* MHNK1 (2%) and surfactin for 4 days (Jakinala et al., 2019).

A very interesting example of the application of biosurfactant in pesticide remediation is from the Patagonia region, which is famous for salmon farming, and to protect salmons from parasitic attack, cypermethrin (A pyrethroid category of pesticide) is used extensively in marine water. Scientists isolated microbial strains *Rhodococcus* species MS13, *Rhodococcus* species MS16, *Pseudomonas* species MS15a, and *Pseudomonas* species MS19 that could degrade cypermethrin by the production of biosurfactant (Aguila-Torres et al., 2020). A novel strain of *Serratia* species Tan 611 has been isolated from Algeria’s oil-contaminated waste-water. Its further sequencing and annotation revealed that it consists of genes that code for catechol 1,2-dioxygenase and naphthalene 1,2-dioxygenase, which are primarily responsible for aromatic derived hydrocarbon catabolism (Clements et al., 2019). An alkane degrading gene Lad-A that codes for monooxygenase have also been identified in the same strain. The bacterially produced biosurfactant has an emulsification index of about 43.47–65.22% and forms biofilms in the presence of oil spills and petroleum. Further studies revealed that *Serratia* species strain Tan611 proves to be one of the best candidates in microbial remediation of aromatic pesticides (Semai et al., 2021). Biosurfactants boost the rate of pesticide degradation when a microbial consortium is used for bioremediation due to the synergistic influence of microbial communities (Purnomo et al., 2017; Bhatt et al., 2019; Femina Carolin et al., 2020).

## Metagenomics: Unraveling the Structure and Composition of Biosurfactant Producing Microbes and Their Role in Pesticide Remediation

Metagenomics analysis based on the sequence and function of the unculturable microbial community will help to uncover information in different ecological niches (Dubey et al., 2019; Kumar et al., 2019; Malla et al., 2019; Kumar and Dubey, 2020). The finding of novel microorganisms or their gene clusters expressing biosurfactants is an example of its application (Datta et al., 2020). Metagenomics provides access to the uncultured microbial population along with their taxonomic and functional composition based on targeted or shotgun sequencing of 16S rRNA regions (Datta et al., 2020). The function-based approach detects and discovers genes capable of forming wholly new bioactive compounds that have never been identified before (Shikha et al., 2021). From pesticide-contaminated materials (soil, water), metagenomics helped create DNA libraries tested for biosurfactant-producing clones. There are many techniques for screening metagenomic libraries for biosurfactants, including function-based approaches like SIGEX (substrate-induced gene expression) and HTP (high-throughput) screening (Datta et al., 2020; Femina Carolin et al., 2020). The investigation of microbial metagenomes can also help researchers to gain a better knowledge of microbes that can produce biosurfactants in a variety of environments, particularly pesticides contaminated soils (Shikha et al., 2021).

**FIGURE 6:**
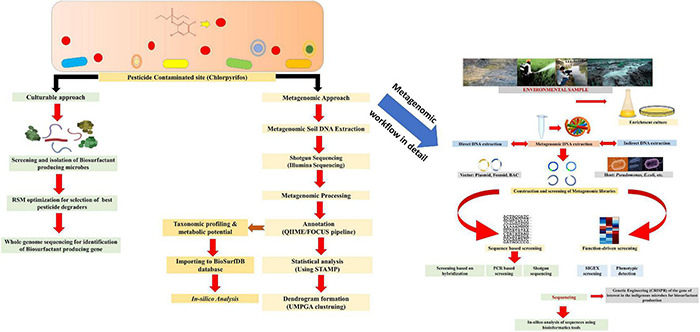
Metagenomics workflow of biosurfactant producing microbes.

The use of function-based metagenomic strategies can be a potent tool in helping to exploit the unique microbial diversity of pesticide-contaminated environments, thereby assisting in the ongoing search for novel biosurfactants with potentially important bioremediation applications (Yadav et al., 2019; Taş et al., 2021). Most research on biosurfactant producing microbes has been limited to soil isolates, primarily from the *Pseudomonas* and *Bacillus* species. However, with the help of metagenomics, it has recently been discovered that a diverse group of soil and marine microbes can produce biosurfactants (Dhanjal and Sharma, 2018; Guerra et al., 2018; Garg et al., 2021) and some of these biosurfactants have shown potential in bioremediation of pesticides (Kennedy et al., 2011; Ghosh and Das, 2018). These microbes include *Azotobacter chroococcum, Cobelia* species, *Myroides* species, *Nocardiopsis alba* MSA10, *Alcanivorax* species, *Micrococcus luteus, Yarrowia lipolytica*. There is a variety of screening approaches for detecting biosurfactant-producing microbes (as discussed in section “Methods for Detection of Microbial Biosurfactants”), some of which could be used for high-throughput (HTP) metagenomic library screening (Domingos et al., 2015; Dhanjal and Sharma, 2018; Guerra et al., 2018). Thus, in screening, it is likely that novel gene clusters involved in biosurfactant production from soil and aquatic microbial assemblages will be discovered, speeding up the development of bioremediation technologies involving biosurfactants in pesticide-contaminated environments (Hemmat-Jou et al., 2018; Taş et al., 2021). The whole workflow of metagenomics investigation of biosurfactant producing microbes is presented in Figure 6.

Metagenomics assists in the investigation of unique biosurfactant-producing genes from the bacteria present in diverse environments and distinct pathways and approaches for improved biosurfactant production. These investigations helped in finding two novel biosurfactants; palmitoyl putrescine and N-acyl amino acids (Jackson et al., 2015; Williams and Trindade, 2017; Hu et al., 2019; Singh et al., 2020b). Given the amount and diversity of biosurfactant synthesizing microbes found in cultured isolates, it is believed that employing metagenomics to investigate the even larger uncultured component of the microbial community will lead to major novel biosurfactant discoveries (Datta et al., 2020; Singh et al., 2020b). A group of researchers identified a new gene involved in biosurfactant synthesis and helps in hydrocarbon degradation. They named it MBSP1 (Carla da Silva Araújo et al., 2020) (metagenomic biosurfactant protein 1) (Araújo et al., 2020). Thies et al. (2016) conducted a metagenomic study by collecting samples from the drain of the slaughterhouse that was rich in microbes belonging to flavobacteriaceae and, through NMR-spectroscopy identified novel biosurfactant as N-acyltyrosines along with N-myristoyl-tyrosine as the dominant species (Thies et al., 2016). Metagenomics delivers an adequate metagenomic database that will give a substantial stock of genes to develop novel microbial strains for targeted application in biosurfactant production and bioremediation (Malla et al., 2018; Datta et al., 2020; Douglas et al., 2020; Kumar et al., 2021b). Metagenomics coupled with bioinformatics removes all the obstacles faced in the process of genomic studies such as phylogenetic analysis, taxonomic profiling, molecular phylogeny, functional characterization of metagenomes, and enzymes and system biology studies, including genetic engineering through CRISPR or TALEN (Singh et al., 2020b). Quite a few bioinformatic pipelines have been developed (Table 2), such as QIIME (quantitative insights into microbial ecology), PICRUSt (phylogenetic investigation of communities by reconstruction of unobserved states), MG-RAST (metagenomic rapid annotations using subsystems technology), Mothur, CLARK, MetaPhlAn2 (metagenomic phylogenetic analysis), MICCA, Metaphyler, MOCAT_2_, TIPP2, mOTU_sv2_, Bracken, etc., for sequence classification and taxonomic profiling of metagenomic data (Liu et al., 2010; Albanese et al., 2015; Truong et al., 2015; Douglas et al., 2020; Singh et al., 2020b). Metagenomics coupled with *in-silico* bioinformatic tools or repositories such as KEGG (Kyoto encyclopedia of genes and genomes), COG (clusters of orthologous groups), EAWAG-BBD pathway prediction system, enviPath, BIOWIN, etc., helps in predictive degradation of pesticides along with the metabolite/biosurfactant identification involved in degradation mechanism (Awasthi et al., 2020; Rodríguez et al., 2020; Shah et al., 2021; Singh et al., 2021). A repository named BioSurfDB (biosurfactant degradation database) consists of about 1,077 microbes, 3,763 genes, 3,430 proteins, and 47 detailed bioremediation pathways using biosurfactants (Araújo et al., 2020; Meenatchi et al., 2020; Kumari and Kumar, 2021).

**TABLE 2:** Bioinformatic pipelines for metagenomic data analysis.

Bioinformatic pipeline	Description	Link	References
Squeeze MATA	Squeeze Meta (A fully automated pipeline) provides multi-metagenome assistance, which allows for the co-assembly of correlated metagenomes as well as the retrieval of specific genomes via binning techniques.	https://github.com/jtamames/SqueezeMeta	Tamames and Puente-Sánchez, 2019
ANASTASIA	ANASTASIA (automated nucleotide amino-acid sequences translational platform for systemic interpretation and analysis) offers a diverse set of bioinformatics toolkits, both publicly available and proprietary, that can be integrated into a variety of algorithmic analytic workflows to perform a variety of data processing applications on (meta)genomic sequence data—sets.	https://galaxyproject.org/use/anastasia/	Koutsandreas et al., 2019
MetaWRAP	MetaWRAP is a shotgun metagenomic data analysis pipeline that starts with raw sequencing reads and ends with metagenomic bins and their analysis.	https://github.com/bxlab/metaWRAP	Uritskiy et al., 2018
WebMGA	It is a customized web server that includes over 20 regularly used functions such as ORF calling, sequence grouping, raw read quality checking, removal of sequencing artifacts and contaminations, taxonomic analysis, functional annotation, and more.	http://weizhong-lab.ucsd.edu/webMGA/ (accessed December 02, 2021)	Wu et al., 2011; Piumini et al., 2021
MetaSUB	Large-Scale Metagenomic Analysis is Made Possible by the MetaSUB Microbiome Core Analysis Pipeline.	https://github.com/MetaSUB/CAP2	Danko and Mason, 2020
MetAMOS	It’s a publicly available, modular metagenomic assembly and analysis pipeline that can help reduce assembly errors, which are prevalent when putting together metagenomic samples, and enhance taxonomic assignment accuracy while lowering computational costs.	https://github.com/treangen/MetAMOS	Treangen et al., 2013; Nathani et al., 2020
SmashCommunity	It is a stand-alone metagenomic annotation and analysis pipeline that works with Sanger and 454 sequencing data. It includes tools for calculating the quantitative phylogenetic and functional compositions of metagenomes, comparing the compositions of several metagenomes, and creating understandable visual representations of such studies.	http://www.bork.embl.de/software/smash/	Arumugam et al., 2010; Sharma et al., 2021
PALEOMIX	PALEOMIX is a modular and user-friendly pipeline that automates the *in-silico* studies behind whole-genome resequencing for modern and ancient genomes.	http://geogenetics.ku.dk/publications/paleomix	Schubert et al., 2014
ARGs-OAP	An integrated structured ARG database is used in an online analytic workflow for detecting antibiotic resistance genes from metagenomic data.	http://smile.hku.hk/SARGs	Yang et al., 2016
HOME-BIO	HOME-BIO (sHOtgun MEtagenomic analysis of BIOlogical entities) is a comprehensive pipeline for metagenomics data analysis that consists of three distinct analytical modules that are meant to analyze big NGS datasets comprehensively.	https://github.com/carlferr/HOME-BIO	Ferravante et al., 2021
QIIME	QIIME is a microbial community analysis software program that has been used to examine and understand nucleic acid data sets from fungal, viral, bacterial, and archaeal populations.	https://qiime2.org/	López-García et al., 2018
MICCA	MICCA is a software pipeline that rapidly integrates quality filtering, clustering of Operational Taxonomic Units (OTUs), taxonomic classification assignment, and phylogenetic tree inference for amplicon metagenomic datasets. It produces reliable findings while maintaining a reasonable balance of modularity and usability.	https://micca.readthedocs.io/en/latest/	Albanese et al., 2015
RIEMS	RIEMS, assigns every individual read sequence inside a dataset taxonomically by cascading different sequence analyses with decreasing stringency of the assignments utilizing multiple software tools. Following the completion of the analyses, the results are reported in a taxonomically ordered outcome procedure.	https://github.com/EBI-COMMUNITY/fli-RIEMS	Scheuch et al., 2015
MG-RAST	MG-RAST is a data platform for processing, analyzing, sharing, and distributing metagenomic datasets that accept open submissions.	https://www.mg-rast.org/	Keegan et al., 2016
PICRUSt	PICRUSt predicts the functional potential of a bacterial community based on marker gene sequencing profiles.	https://github.com/picrust/picrust2	Douglas et al., 2020
MetaPhlAn	MetaPhlAn (Metagenomic Phylogenetic Analysis) is a program that uses metagenomic shotgun sequencing data to profile the makeup of microbial communities. It depends on 17,000 reference genomes to identify unique clade-specific marker genes.	https://huttenhower.sph.harvard.edu/metaphlan2/	Truong et al., 2015
FMAP	FMAP (*F*unctional *M*apping and *A*nalysis *P*ipeline) is an open-sourced, stand-alone functional analysis pipeline for analyzing whole metagenomic and meta transcriptomic sequencing data.	https://github.com/jiwoongbio/FMAP	Kim et al., 2016
TIPP2	It is a marker gene-based abundance profiling method that controls classification precision and recall by combining phylogenetic placement with statistical methodologies. Over the original TIPP technique, it includes an updated set of reference packages and various algorithmic advancements.	https://github.com/smirarab/sepp/blob/tipp2/README.TIPP.md	Shah et al., 2021

To date, only a few research employing genetic modification methods for biosurfactant production have been published, and one such research is genetic modification of wild *Bacillus* strain for surfactin production (Tsuge et al., 2001). *Bacillus* species are engineered to increase their production through operon promoter transfer (SrfA) or upregulation of the exporter (YerP). Due to intricate metabolic regulation and its long genomic sequence, this operon’s production is difficult (Hu et al., 2019; Singh et al., 2020a). However, genetic engineering methods only resulted in a few or single-gene alterations, and commercial manufacturing of biosurfactants has yet to be achieved. As a result, experimentation-based optimizations to synthesize biosurfactants are still ongoing, and a new regulatory aspects need to be investigated, and the latest CRISPR based methods should be used to transfer biosurfactant producing genes to indigenous microbes residing in contaminated sites (Mulligan, 2009; Sekhon et al., 2011; Soares da Silva et al., 2019; Kumar and Dubey, 2020).

## Conclusion and Prospects

Pesticides are complex compounds that are hydrophobic. When used in excess, pesticides pollute the air, soil, and water bodies because they interact with soil particles and leach deep into the soil and water bodies, making them inaccessible for microbial activity to thrive. Microbial biosurfactants serve an essential role in making these pesticides accessible for microbial enzymatic breakdown. Biosurfactants dissolve pesticides linked to soil particles and create emulsions at and above their CMC, thus increasing the bioavailability of the pesticide molecule in the soil. Different microbes secrete different categories of biosurfactants, each of which contributes more or less to enhancing the remediation process. More emphasis should be placed on improving process parameters to maximize the production of biosurfactants and their application in pesticide remediation. Tapping the potential of biosurfactant producing microbes by using the latest omics platform and gene-editing tools may offer a sustainable way to remediate these pesticides from the environment.

Although, research is going on for the production of biosurfactants from microbes. But still, many areas remain unexplored and need further investigation, such as:

• Most of the microbial biosurfactants have anti-microbial activities and are not suitable for remediation studies as this may harm the remediation process instead of enhancing it.
• Pesticide manufacturers must switch to biosurfactants instead of synthetic surfactants used as emulsifiers as they are highly toxic and persistent in the environment.
• Metagenomics coupled with DNA-stable isotope probing can be used in future studies to identify novel microbes with biosurfactant producing potential as it overcomes the impediment faced in functional screening using metagenomics.
• Metagenomics studies and other omics and *In-silico* studies need to enhance access to biosurfactants producing microbes from highly contaminated habitats or high-stress conditions such as high pH, temperature, and salinity, etc.
• More genetic and bioengineering studies need to be conducted to identify genes involved in biosurfactant production and the implementation of advanced CRISPR (clustered regularly interspaced short palindromic repeats) technology to enhance biosurfactants’ production.
• Identification of biosurfactant genes and their incorporation into microbial species commonly found in contaminated sites utilizing the CRISPR tool which will enhance the process of pesticide remediation.

## Author Contributions

AR prepared the draft of the manuscript under the supervision and guidance of AK. AK and JD revised and edited the manuscript. All authors contributed to the article and approved the submitted version.

## Conflict of Interest

The authors declare that the research was conducted in the absence of any commercial or financial relationships that could be construed as a potential conflict of interest.

## Publisher’s Note

All claims expressed in this article are solely those of the authors and do not necessarily represent those of their affiliated organizations, or those of the publisher, the editors and the reviewers. Any product that may be evaluated in this article, or claim that may be made by its manufacturer, is not guaranteed or endorsed by the publisher.
